# One System for All: Is Mass Spectrometry a Future Alternative for Conventional Antibiotic Susceptibility Testing?

**DOI:** 10.3389/fmicb.2019.02711

**Published:** 2019-11-26

**Authors:** Martin Welker, Alex van Belkum

**Affiliations:** Microbiology Research Unit, BioMérieux SA, La Balme-les-Grottes, France

**Keywords:** antibiotic susceptibility, diagnosis, clinical microbiology, mass spectrometry, MALDI-TOF MS

## Abstract

The two main pillars of clinical microbiological diagnostics are the identification of potentially pathogenic microorganisms from patient samples and the testing for antibiotic susceptibility (AST) to allow efficient treatment with active antimicrobial agents. While routine microbial species identification is increasingly performed with matrix-assisted laser desorption/ionization time-of-flight mass spectrometry (MALDI-TOF MS), routine AST still largely relies on conventional and molecular techniques such as broth microdilution or disk and gradient diffusion tests, PCR and automated variants thereof. However, shortly after the introduction of MALDI-TOF MS based routine identification, first attempts to perform AST on the same instruments were reported. Today, a number of different approaches to perform AST with MALDI-TOF MS and other MS techniques have been proposed, some restricted to particular microbial taxa and resistance mechanisms while others being more generic. Further, while some of the methods are in a stage of proof of principles, others are already commercialized. In this review we discuss the different principal approaches of mass spectrometry based AST and evaluate the advantages and disadvantages compared to conventional and molecular techniques. At present, the possibility that MS will soon become a routine tool for AST seems unlikely – still, the same was true for routine microbial identification a mere 15 years ago.

## Introduction

Within a few years mass spectrometry (MS) as a tool for microbial identification replaced conventional identification techniques based on patterns of substrate utilization in many diagnostic laboratories world-wide (van Veen et al., 2010; Patel, 2015). The two major commercially available systems, namely MALDI Biotyper (Bruker Daltonik, Bremen, Germany) and VITEK MS (bioMérieux, Marcy l’Étoile, France) are continuously extended in their range of identifiable taxa, which include Gram-negatives, Gram-positive cocci, mycobacteria, non-fermenters, anaerobes, yeasts, molds, and others, primarily focusing on microorganisms of medical and industrial relevance (Welker et al., 2019). Numerous studies highlight that the technology is not limited to this but principally suitable to rapidly identify any microbial species with only minimal and relatively uniform sample preparation procedures – given respective reference data are available (Welker, 2011). This potential of universal application raised the question whether MS could also play a prominent role in antibiotic susceptibility testing (AST) or antibiotic resistance testing (ART) by faster and possibly cheaper procedures compared to conventional tests. This sparked a number of studies and publications (Hrabák et al., 2013; Burckhardt and Zimmermann, 2018; Oviaño and Bou, 2019), proposing multiple approaches that will be succinctly discussed in the following and will be evaluated for their potential to – at least partly – replace conventional AST in the future. Further, we consider that the fields of AST and ART are developing dynamically with a wide variety of new techniques on the horizon and MS is only one technology among others (van Belkum and Dunne, 2013).

MS either targets susceptibility or resistance of a clinical isolate. With ART the presence of biomarkers – proteins, carbohydrates, lipids, enzymatic activity – for a particular resistance mechanism is detected, expectedly adopted to different types of clinical specimens and resistance mechanisms [for an overview on the latter, see, for example, Blair et al. (2015), Cowen et al. (2015), Yelin and Kishony (2018)]. This is fundamentally different to conventional AST where no particular biomarkers are detected but the response, or the lack thereof, of living organisms upon exposure to various antibiotics. Generally, ART tells the clinician which antibiotics will not be effective to treat a patient, without directly suggesting which antibiotics presumably would be effective, while AST allows to directly select antibiotics for effective treatment. Nevertheless, the identification of a particular resistance mechanism can help direct treatment options. For example, carbapenem resistance can be due to a modification in drug influx (like porin mutants; Livermore, 2001) or it can be due to the presence of carbapenemases: only the latter resistance mechanism could be overcome in therapy by inhibitors such as clavulanic acid (Nordmann et al., 2012).

In the last decade a number of studies have been published, suggesting the use of matrix-assisted laser desorption/ionization time-of-flight MS (MALDI-TOF MS), liquid chromatography-MS (LC-MS; in various forms) or other MS technologies for the rapid antibiotic resistance or susceptibility testing of clinical isolates. Most approaches can be classified as summarized in Table 1.

**TABLE 1:** Principal assays to determine resistance or susceptibility of microbes by mass spectral techniques.

**Assay principle**	**R/S**	**Technique**	**Targeted resistance mechanism**	**Targeted taxa and prerequisites**
Identification of resistant phylogroups	R	WCMS	taxon-specific	Selected taxa for which molecular reference data are available
Direct detection of proteins conferring resistance	R	MALDI MS LC-MS	General, but with largely technical limitations	General, but protein sequences have to be known
Detection of biomarkers associated with antibiotic resistance	R	MALDI MS	Specific for selected mechanisms	Individual taxa, preferably with sufficient genomic or PCR data
Monitoring antibiotic degradation	R	MALDI MS LC-MS	Lactamases (carbapenemases)	Possibly general; explored mostly for Enterobacteriaceae, *Pseudomonas*, *Acinetobacter*
Detection of microbial growth	S	MALDI MS	General	General for taxa growing in homogeneous suspension

*In column R/S it is indicated whether resistance (R) or susceptibility (S) is determined.*

## Identification of Resistant Phylogroups by Wcms

WCMS (whole-cell mass spectrometry) refers to the analysis of whole cells or crude, unfractionated extracts of whole cells, by MALDI-TOF MS which is the technique that is applied for MALDI-TOF MS based microbial identification, most often in a mass range of 2000–20000 Da and by using α-Cyano-4-hydroxycinnamic acid (CHCA) as matrix (Fenselau and Demirev, 2001; Sauer and Kliem, 2010; Welker, 2011).

Soon after microbial identification by WCMS became a serious perspective in clinical diagnostic, the differentiation of methicillin-resistant and methicillin susceptible *Staphylococcus aureus* (MRSA and MSSA, respectively) by WCMS was proposed. The global population of *S. aureus* is organized in clonal complexes (CC) that represent distinct sub-specific taxonomic units that can be differentiated based on multi-locus sequence typing (MLST) of a set of housekeeping genes (Feil et al., 2004). Individual CCs comprise multiple sequence types (ST). Resistance to methicillin, in contrast, is generally acquired by horizontal gene transfer and the genetic element coding for the resistance is the *Staphylococcus* chromosomal cassette carrying *mec* genes (SCC*mec*) coding for the Penicillin-binding-protein 2a (PBP2a, ca. 70 kDa; Enright et al., 2002). SCC*mec* is a mobile genetic element that can be transferred from a donor to a recipient strain, independent of the CC to which both belong. Therefore, within each CC strains with (MRSA) and without resistance (MSSA) to methicillin exist (Chambers and DeLeo, 2009).

A pioneering study on discrimination of MRSA and MSSA with WCMS by Edwards-Jones et al. (2000) was confirmed by other studies (Walker et al., 2002; Muroi et al., 2012) while differentiation failed in others (Bernardo et al., 2002; Schlebusch et al., 2010). This apparent inconsistency was eventually explained by Josten et al. (2013) who could show that WCMS could reproducibly differentiate certain CC of *S. aureus* irrespective of the individual strains’ resistance status. This is in agreement with a study by Szabados et al. (2012) who analyzed a pair of isogenic SCC*mec*-harboring and SCC*mec*-lacking strains and found no difference in peak profiles. Since the prevalence of resistant and susceptible clones is highly variable among individual CCs, the earlier and optimistic studies were strongly dependent on the selection of analyzed strains, this is MRSA from one CC and MSSA from another. The differentiation of CCs or sequence types irrespective of resistance status is, nonetheless, been considered as a valuable tools for a rapid epidemiological evaluation of clinical isolates (Østergaard et al., 2015; Camoez et al., 2016; Østergaard and Møller, 2018), potentially tracing individual clones such as the notorious USA300 (Boggs et al., 2012). A reliable identification of (certain) MRSA was found to be possible with a simple WCMS approach by focusing on individual marker peaks related to the resistance conferring genetic elements (Josten et al., 2014) and next section). Detecting methicillin resistance *per se* by WCMS has to be deemed unsuccessful at the present stage.

**FIGURE 1:**
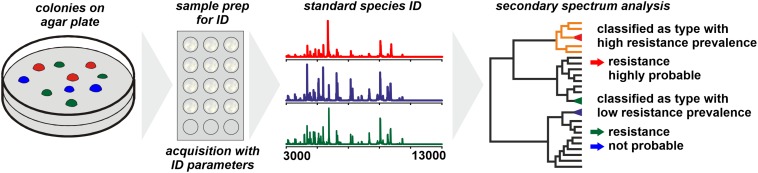
Presumptive assessment of the resistance status of a microbial sample based on phylotyping by MALDI-TOF MS. The prerequisite is a comprehensive database on the prevalence of particular resistance mechanisms among individual phylotypes.

For *Bacteroides fragilis* the WCMS approach was successful in differentiating carbapenem-resistant from susceptible isolates (Nagy et al., 2011; Wybo et al., 2011). The resistance to carbapenems (and other β-lactams) in *B. fragilis* is conferred to a gene – *cfiA* – the distribution of which among *B. fragilis* isolates is largely consistent with phylotypes with isolates belonging to Division II generally harboring the (silent) *cfiA* gene (Litterio et al., 2017). The corresponding protein, CfiA, has a mass of some 25,200 Da which is generally beyond the spectrum acquisition mass range in WCMS. Therefore, by WCMS actually not the resistance of an isolate is detected but the phylotype that is associated with an elevated prevalence of carbapenem resistance (Figure 1; Cordovana et al., 2018). WCMS has been used to screen large sets of isolates to reveal the regional prevalence of resistant isolates (Jeverica et al., 2019) as well as dynamic changes over time (Ferløv-Schwensen et al., 2017).

Similar approaches to identify vancomycin resistant *Enterococcus* sp. (VRE, VanA positive) showed mixed results. While Griffin et al. (2012) and Nakano et al. (2014) found that VRE and non-VRE could be distinguished by WCMS, Nowakiewicz et al. (2017) found only a weak correspondence of MALDI fingerprint clustering to molecular typing. As with CfiA, VanA (ca. 37,400 Da) itself has not been detected in the studies. Nonetheless, WCMS can be used to trace outbreaks of VRE in certain specific clinical settings (Holzknecht et al., 2018).

For Enterobacteriaceae and *Pseudomonas aeruginosa*, no specific peak patterns to reliably differentiate ESBL-positive and ESBL-negative isolates were found (Schaumann et al., 2012).

The inconsistency in the publications on this matter – which is, not unusual, most likely biased by the scarcity of publications with negative or inconclusive results – is explained by the evolutionary context of acquisition of resistance.

When individual strains are analyzed by WCMS the observed peak patterns largely represent products of housekeeping genes and structural proteins while proteins conferring antibiotic resistance (lactamases, PBP2a, etc.) have molecular weights generally well beyond 20,000 Da. Hence, by WCMS generally antibiotic resistance biomarkers are not directly detected (see next section). Instead, phylotypes are identified with specific prevalence of resistant clones among them and for a meaningful interpretation of results, the phylotype specific prevalence needs to be known (Figure 1). Most genes coding for acquired resistance mechanisms are disseminated through horizontal gene transfer (Juhas, 2015; von Wintersdorff et al., 2016), a process supposedly enhanced by the presence of antibiotics (Jutkina et al., 2018). In species that are organized mostly clonal and where the acquisition of a particular resistance mechanism is known to be a rare event, a stable and consistent correlation of WCMS peak pattern to resistance state could be expected and explored for clinical diagnostics. Since this is generally specific for individual taxa, an established typing method for one taxon cannot be transferred to other taxa without further investigation.

Therefore, a WCMS approach that may yield rapid and accurate results with respect to antibiotic resistance for one species may be unreliable for others. In the development phase of a test, an inadequate selection of isolates bears the risk of a biased over-interpretation, like in some reports on identification of MRSA. Further, the possibility of specific local occurrence of phylotypes and resistance traits needs to be considered: while at one location a particular species or sub-specific type may be represented only by resistant clones, at other locations both, resistant and susceptible clones co-exist. Hence, the portability of respective diagnostic MS methods from one site to others can be curtailed. A thorough validation and development of site-specific reference spectrum database is therefore highly recommended.

Despite all limitations discussed, once established and validated – possibly only for a local setting – the method can be a valuable analytical tool for the early recognition of potentially resistant isolates. The major advantage being the fact, that spectra acquired for the purpose of identification can be directly used for a secondary data analysis without the need for a repeated sample preparation.

## Detection of Proteins Conferring Resistance

As many resistance mechanisms are based on the expression of particular proteins or on particular modifications of common proteins, the idea of proteomics as a tool to detect antibiotic resistance is nearly self-evident (Pérez-Llarena and Bou, 2016).

**FIGURE 2:**
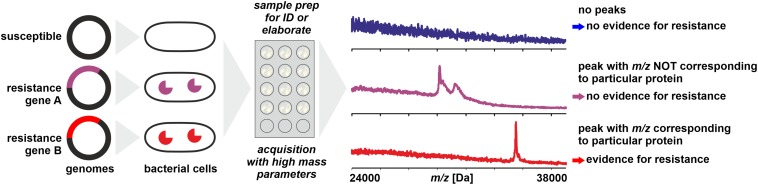
Direct detection of proteins conferring resistance (e.g., lactamases) from microbial cells by MALDI-TOF MS. Sample preparation can be simple or elaborate as mentioned in the text. Peaks with *m/z* not corresponding to the theoretical mass of the targeted protein (± tolerance allowed for analytical error) do not allow conclusions on the resistance status of the isolate.

The most straightforward approach for resistance testing would be the direct detection of resistance proteins like lactamases by WCMS (Figure 2). Lactamases occur in multiple types and with a high sequence variability and range in molecular mass from some 25 to 40 kDa (Ambler, 1980; Bush and Jacoby, 2010). With MALDI-TOF MS, proteins of masses exceeding hundreds of kDa can be detected – but generally only following purification and concentration steps. When whole cells of microorganisms are analyzed in a range of *m/z* 2,000–20,000, for the majority of samples, intense peaks beyond *m/z* 13,000 are rarely recorded. There are several reasons why certain large proteins (>20 kDa) – that are undoubtedly present in the cells – are difficult to be detected with WCMS:

• Larger molecules produce smaller peaks because the ion signals are distributed on an increasing number of isotopic peaks (primarily due to the natural occurrence of ^13^C). Hence, for exactly the same number of ions of a small and a large protein, the peak height is lower for the large protein while peak areas are (theoretically) the same (Horn et al., 2000). The increased abundance of isotopic peaks with increasing molecular mass also causes a decline in mass precision for larger molecules (Stump et al., 2003).
• Proteins with enzymatic functions are generally less abundant in living cells than structural proteins such as ribosomal proteins (Pineda et al., 2003). Enzymes, including ones that confer antibiotic resistance, generally show a higher variability in conditional expression levels compared to structural proteins (Bandow et al., 2003).
• Further, the poor detectability of large proteins can be linked to their location in the cells (for example in membranes), their poor co-crystallization with standard matrix solutions (very hydrophobic proteins), formation of quaternary structures that decreases detectability, lower stability in the MALDI process, and others.

Nevertheless, proteins with molecular weights exceeding 20 kDa have been detected directly from preparations of whole bacterial cells. For example, cell wall proteins of *Clostridioides difficile* (∼ 30–39 kDa) can be used to type isolates of this species (Rizzardi and Åkerlund, 2015) and to follow outbreaks (Ortega et al., 2018).

Direct detection of resistance enzymes like β-lactamases by WCMS has been reported by Camara and Hays (2007) but this concerned an *in vitro* transformed strain of *E. coli* that apparently hyper-expressed the β-lactamase (*m/z* ≈ 29,000) in the presence of antibiotics. In the absence of antibiotics the β-lactamase could not be detected. With non-transformed, resistant strains the detection of β-lactamases was not successful by simple sample preparation procedures, most likely due to a lower – and natural - rate of protein expression (Schaumann et al., 2012). A more elaborate sample preparation of periplasmic proteins allowed the detection of CMY-2 cephalosporinases (*m/z* 39,850) in various Enterobacteriaceae, however, not in all strains positive for CMY-2 by reference methods (Papagiannitsis et al., 2014). With a sample preparation using detonation nanodiamonds, Chang et al. (2018) were able to detect carbapenemases in *Acinetobacter baumannii* isolates (*m/z* ∼40,279 ± 87), although 4 out of 15 susceptible strains showed also a signal in this mass range. With a moderately complex sample preparation procedure, Espinosa et al. (2018) achieved the detection of CMY-2 carbapenemases (*m/z* 39,800) in *E. coli* by MALDI-TOF MS. This method also allowed the detection of mature KPC-2 β-lactamase (*m/z* 28,544) in positive blood cultures (Espinosa et al., 2018).

Besides the difficulties mentioned above to detect a particular protein that is expressed at a comparatively low level, it needs to be considered that with simple MALDI-TOF MS (e.g., no tryptic digest and database search with Mascot software; Perkins et al., 1999) only an *m/z* value is obtained that does not allow an unambiguous identification of a protein. In the case of β-lactamases, where a large number of variants are known with varying molecular mass, this means that a positive identification can only be achieved when the theoretical, *in vivo* value of *m/z* of a strain’s β-lactamase is known, taking into account possible post-translational modifications. For example, mature TEM variants described for *Klebsiella pneumoniae* span over a theoretical mass range of at least *m/z* 28,838 to 28,954 (sequences obtained from GenBank and processed with SignalP 5.0; Armenteros et al., 2019). Therefore, without prior knowledge of the *in vivo* mass of a particular target protein a peak in the mass range where most of the β-lactamases are expected allows only a tentative conclusion that this peak indeed could represent a β-lactamase. Strictly speaking, the detection of a lactamase in a bacterial clone by WCMS can only be considered valid, when the expected peak’s *m/z* can be derived from the clone’s genome sequence and known post-translational modifications – which would make a mass spectral analysis as diagnostic tool dispensable.

Even with the molecular mass known, a peak with a matching *m/z* is by no means a prove of its identity because a large number of unrelated proteins can fall within the same, narrow *m/z* window. The unambiguous assignment of peaks in a (linear) MALDI-TOF mass spectrum of a microbial proteomic extract is still a major challenge and even with sophisticated instrumentation, an identification by top–down proteomics (Catherman et al., 2014) is limited to *m/z* < 15,000 (Fagerquist, 2017).

For these reasons, detection of antibiotic resistance through detection of lactamases or other resistance conferring proteins may not develop into a widely applied method for microbial diagnostics.

**FIGURE 3:**
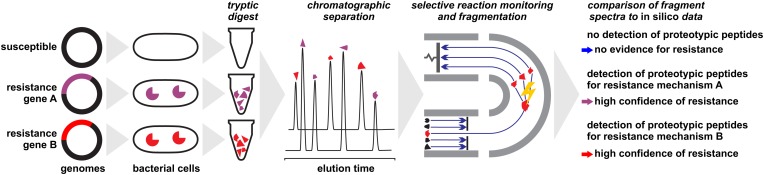
Detection of proteins conferring antibiotic resistance by liquid-chromatography/mass spectrometry approaches. Protein extracts of microbial cells are subjected to a tryptic digest before analyzed. In a final step, fragment patterns of proteotypic peptides are compared to *in silico* sequences for protein identification or confirmation, respectively.

The problem of peak ambiguity can be overcome by applying more elaborate sample preparation procedures and MS technology (Figure 3). Subjecting a protein extract to a tryptic digest and subsequent analysis with LC-MS/MS followed by sequence analysis and database search, for example, allows the unambiguous detection of many proteins (Reiter et al., 2011; Picotti and Aebersold, 2012; Gillet et al., 2016), including those conferring resistance through alteration of drug targets such as PBP2a in MRSA (Charretier et al., 2015a). Several chromatographic and electrophoretic methods in combination with different MS techniques have been successfully applied in this field. Analysis of proteotypic peptides by LC-ESI-MS/MS allows the identification of modified porins (Opr) conferring multi-drug resistance and AmpC conferring resistance to cephalosporins and antipseudomonal penicillins (Charretier et al., 2015b). The identification of *Klebsiella pneumoniae* carbapenemase (KPC) within less than 2 h has been proposed by LC-MS/MS detection of three peptide markers (Wang et al., 2017). A capillary-electrophoresis ESI-MS/MS method was shown to be able to establish the presence of OXA-48 and KPC in carbapenemase positive isolates, independent of species and degree of susceptibility by detecting only 10–17 specific peptides (Fleurbaaij et al., 2014). The same family, OXA-48 was targeted by Strich et al. (2019) who exploited a protein sequence database for an *in silico* identification of core peptides. The vast diversity of β-lactamases, with thousands of individual proteins (Bush, 2018), require profound *in silico* analyses to ensure that the selected marker peptides truly are representative.

These techniques also offer the possibility to analyze a sample for multiple resistance proteins simultaneously. For example, Cecchini et al. (2018) established a method to detect 16 proteins associated with antibiotic resistance, including lactamases and efflux systems, in a single run. A similar method based on nano-LC-MALDI-MS/MS allows the detection of marker peptides for various β-lactamases and other resistance proteins (Hart et al., 2015).

The detection of an individual resistance protein by LC-MS/MS [or gel electrophoresis (GE)-MS/MS] generally allows accurate predictions on the resistance status of isolates, especially when multiplex methods are applied. The realization of respective analyses, however, requires substantial investment in high-end instrumentation, profound chemical expertise, and a validation for each individual protein family. The effort for the latter may be reduced with *in silico* studies, by which tryptic fragments with conserved amino acid sequences shared by multiple protein variants (i.e., individual peptides of the TEM, VIM, or CMY families) can be identified and used as biomarkers for protein families instead of individual proteins (Mesuere et al., 2016).

Fundamentally, the amino acid sequence of a resistance protein needs to be known to allow for accurate detection. With the vast and increasing amount of genomic sequence data, the major limiting factor may be the resources needed for bioinformatic analyses (Scholz et al., 2012), especially when newly described protein families are targeted for which the global diversity is difficult to estimate. For example, a PBP 2a variant (MecC; first described in emerging MRSA in Scandinavia; García-Álvarez et al., 2011), shares only <65% identity (<80% positives) with MecA and is most likely not detected by a method targeting MecA while it still renders a clone methicillin-resistant.

Another prerequisite for a successful analysis is the expression of resistance conferring proteins under the chosen culture conditions and consequently their presence in the proteome in detectable amounts. The induction and expression of proteins such as lactamases is often specific for particular genes and particular taxa (Bennett and Chopra, 1993) and not fully understood yet (Li et al., 2016; Palmer et al., 2018). For example, inducible AmpC mediated resistance in Enterobacteriaceae occurs in primarily in isolates with a chromosomally encoded and intact *amp*-operon where the expression of AmpC is often triggered by lactam antibiotics (Tamma et al., 2019). Thus, a negative proteomic result for a lactamase protein does not exclude the possibility that the protein gene was simply not expressed or only at low levels under the given conditions and hence the isolate’s susceptibility or resistance level cannot be deduced.

On the other hand, proteomic approaches are widely applicable and not confined to a single or a few resistance mechanisms: proteins responsible for antibiotic degradation, modified porins or altered peptidic target molecules can be detected in the same workflow. It is therefore fairly safe to state that any antimicrobial resistance mechanism involving particular proteins can be detected by LC-MS/MS and related techniques.

With respect to the workflow, neither MALDI-TOF MS nor LC-MS/MS approaches have been integrated into routine analysis in clinical microbiology laboratories nor transformed to a commercial product yet. The time to result for individual tests is reported to be in the range of a few hours.

## Detection of Biomarkers Associated With Antibiotic Resistance

**FIGURE 4:**
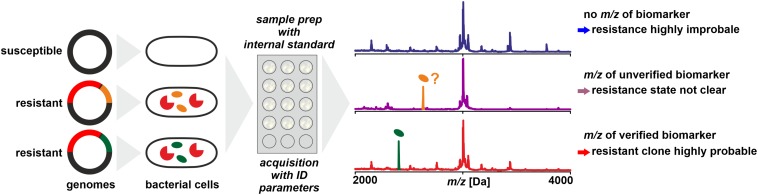
Detection of biomarkers related to antibiotic resistance by MALDI-TOF MS, for example, by being co-coded on the same mobile genetic element as the resistance conferring protein. Biomarker peaks have generally lower *m/z* compared to the resistance proteins. As a prerequisite, the biomarker has to be well characterized and the relationship of biomarker and resistance protein is established by molecular analyses and/or by analyzing a meaningful number of reference samples.

It is not necessarily the resistance conferring protein that is detected by MS – with the limitations discussed above – to deduce an isolates’ resistance status. In a number of cases it could be shown that biomarkers in mass spectra acquired for routine identification by WCMS are indicative of certain resistance types (Figure 4). These are either proteins or peptides co-coded with the protein actually responsible for resistance or modified target molecules that render antibiotics inefficient.

### Co-expressed Proteins

In a study on determination of methicillin resistance by WCMS, Du et al. (2002) analyzed MRSA and MSSA isolates in a mass range of 500 – 3000 Da, i.e., in a mass range not generally used for routine identification. In this mass range, they claimed to have identified biomarkers indicative of MRSA without further characterizing them. Later, Josten et al. (2014) identified one of the peaks (*m/z* 2415) as phenol-soluble modulin (PSM-mec). This peptide is coded in certain types of the SCC*mec* element, together with the PBP2a which confers resistance by a lowered affinity to methicillin. The detection of the biomarker proved to be highly specific – but only for isolates harboring SCC*mec* types II and III while generally lacking in isolates harboring SCC*mec* types I, IV, or XI (Rhoads et al., 2016). Therefore, while the presence of a certain peak in a spectrum is indicative of MRSA, its absence must not be interpreted as indication of MSSA (Schuster et al., 2018). Differences in peak patterns in this lower mass range can be used to type MRSA isolates for epidemiological purposes (Lindgren et al., 2018).

A similar biomarker, associated with *K. pneumoniae* carbapenemase was identified by Lau et al. (2014). The peak at *m/z* ∼11,109 could be assigned to an associated protein encoded on a plasmid that contains the KPC-gene (*bla*_*KPC*_). In a larger set of samples, the consistent presence of the biomarker peak could be verified, but only in isolates that harbor plasmids with a particular transposon (Tn4401a) while absent in KPC producing strains with other transposon types (Youn et al., 2016). This finding was further confirmed to be independent of the sequence type of *K. pneumoniae* (Gaibani et al., 2016) and isolates resistant due to other carbapenemases (VIM and NDM, for example) were found negative (Centonze et al., 2018). Therefore, the reported specificities and sensitivities are possibly only valid for the tested sample sets. The presence of the peak in a spectrum indicates a KPC producing isolate harboring a specific plasmid while its absence doesn’t allow a conclusion whether the isolate produces carbapenemases or not.

### Modified Target Molecules

Another way to look at resistance of an isolate toward a particular antibiotic or class of antibiotics is to analyze target molecules a modification of which can confer antibiotic resistance. In MRSA this could be PBP2a. These enzymes, a subgroup of the transpeptidase family, are essential for bacterial cell wall synthesis. Yet, as discussed above, the size of PBP2a makes it inaccessible to rapid and simple mass spectral analysis.

Other modified target molecules are, for example, individual ribosomal proteins making the clones resistant to antibiotics that inhibit protein biosynthesis, e.g., streptomycins. However, only few examples are known such as *E. coli* with a deletion in 30S ribosomal protein S7 (Wilcox et al., 2001), a Lys→Arg mutation in 30S ribosomal protein S12 in *Pantoea vagans* (Rezzonico et al., 2010), or a Thr→Pro mutation in 30S ribosomal protein S5 in spectinomycin resistant *Neisseria gonorrhoeae* strains (Ilina et al., 2013). The scarcity of examples is not very surprising considering the central role of highly conserved ribosomal proteins in basic cell physiology which makes most if not all mutations potentially deleterious. However, mutations in ribosomal proteins leading to resistance are supposedly detectable by rapid and simple mass spectral analysis.

Another target molecule that gained the attention of clinical mass spectrometrists is lipid A, a conserved part of the lipopolysaccharide (LPS) molecules of which the cell membrane of (primarily) Gram-negative bacteria consists (Raetz and Whitfield, 2002; Wang and Quinn, 2010). The interaction of polymyxin antibiotics with LPS is reduced when lipid A is modified by substitution of at least one phosphate group (Moskowitz et al., 2004; Pelletier et al., 2013) by phosphoethanolamine (PEA) via a plasmid encoded PEA transferase (Liu et al., 2017). These modifications in lipid A molecules can be detected as mass shifts compared to peaks of wild-type lipid A in mass spectra, for example, from *m/z* 1796 to 1919 in *E. coli*, *m/z* 1824 to 1947 in *K. pneumoniae* or *m/z* 1910 to 2033 in *A. baumannii* (Liu et al., 2017).

The modification of lipid A confers resistance to colistin, one of the important last resort antibiotics used today (Dortet et al., 2018a). Presumably, the wide use of colistin in production animals for decades, has fueled the wide spread of resistance mechanisms causing severe problems in clinical therapy (Catry et al., 2015). The standard procedure for detection of colistin resistance, broth microdilution (EUCAST, 2016), is comparatively slow, not very reproducible, and laborious (Poirel et al., 2017) and therefore a rapid mass spectral approach is attractive. The mass spectral analysis requires an adapted extraction and hydrolyzation procedure (El Hamidi et al., 2005; Larrouy-Maumus et al., 2016; Liang et al., 2019) and the MALDI-TOF MS analysis has to be performed preferably in negative ion extraction mode, generally in the range of *m/z* 1000–3000 (Dortet et al., 2018a). First, clinical studies with larger sets of clinical isolates of *E. coli* (Dortet et al., 2018a), *A. baumannii* (Park et al., 2018), and *Acinetobacter* spp. (Leung et al., 2018) showed promising results in terms of sensitivity and specificity but with some inconsistencies that raised questions on possible hetero-resistance (Park et al., 2018). Due to the high variability of lipid A structures among bacterial species of clinical relevance (Trent et al., 2006), for a mass spectral assay target molecules need to be identified for individual species. Further, the possibility that individual strains may produce multiple variants of lipid A has to be taken into account (Darveau et al., 2004).

When the detection of resistance biomarkers by MS is technically feasible with moderate sample preparation requirements, such assays may be an interesting first-line analytical tool for an efficient classification of resistant isolates. Care must be taken to not over-interpret the test results (i.e., presence or absence of a particular peak in spectra), firstly, because of technical issues: MS, in particular MALDI-TOF MS produces spectra with an intrinsic variability due to factors such as analyte-matrix co-crystallization processes and desorption and ionization efficiency, for example, rendering low intensity peaks prone to inconsistent recording.

Secondly, it has to be acknowledged that an *m/z* value obtained with linear MALDI-TOF MS, even a highly accurate one, does not allow for unambiguous assignment of a peak to a particular protein. In most species, a very large number of proteins can be found with theoretical masses in a narrow mass window. Therefore, a biomarker peak for resistance should produce peaks of moderate to high intensity in a mass range that is not densely populated with other, intense peaks. Equally, a single amino acid exchange in a biomarker molecule can result in a mass shift, that may suggest the absence of the target protein and hence produce a false-negative result.

Thirdly, the biological background has to be taken into account, in particular, the conservation of the biomarker, i.e., the amino acid sequence and post-translational modifications in proteins, its stable association to individual resistance mechanisms, and its stable expression or biosynthesis, respectively. As discussed above, the biomarkers for MRSA and KPC give valid results only for specific types of resistant clones and negative results must not be interpreted as lack of resistance.

If these limitations are considered, detection of resistance biomarkers by MS can produce relevant results with only moderate efforts in a clinical context. Possibly, respective results can be directly used to adjust patient treatment, primarily by avoiding futile treatment with an ineffective antibiotic – and the imminent loss of critical time – but also to support to streamline further analyses.

## Monitoring Antibiotic Degradation

**FIGURE 5:**
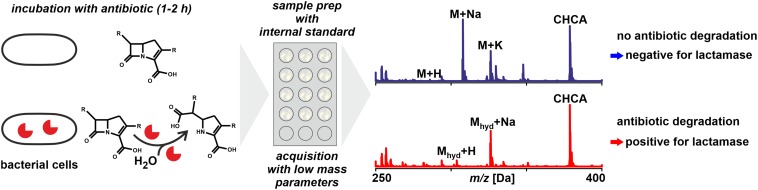
Monitoring antibiotic degradation to detect lactamase activity in microbial samples by MALDI-TOF MS. For particular species and types of lactamases the incubation time may require extension avoid false negatives. The technique is in principle applicable to other resistance mechanisms that involve a detectable modification of antibiotics.

Another strategy to analyze clinical isolates for antimicrobial resistance makes use of MALDI-TOF MS as analytical tool to follow antibiotic degradation (Figure 5). In this approach, a bacterial isolate suspected of carrying an antibiotic degradation enzyme is exposed to substrate antibiotic in a suspension. The preparation of the suspension in most studies generally follows a very simple procedure: a loop-full of bacterial cells are transferred to a reaction tube containing a small volume of the medium (in a range of 10–500 μL) and antibiotics in a concentration that allows a direct detection by MALDI-TOF MS without the need of a concentrating step. Cells are suspended with the loop, or vortexed if needed, and incubated for a pre-defined time at an appropriate temperature. Samples were collected after centrifugation and transfer of a small volume of supernatant to a spot on a MALDI-target plate. With MALDI TOF MS analyses the kinetics of antibiotic metabolization is recorded and interpreted. This principle has been followed primarily for β-lactamase activity that leads to the hydrolyzation of the β-lactam moiety (and possibly further degradation) that results in changing ratios of peak heights of substrate and product (Burckhardt and Zimmermann, 2011; Hrabák et al., 2011, 2012; Hooff et al., 2012; Sparbier et al., 2012; Carvalhaes et al., 2013; Mirande et al., 2015).

In principle, any resistance mechanism that inactivates an antibiotic through a chemical derivatization resulting in peak shifts in mass spectra can be detected. The method discussed in the following is largely focused on the detection of carbapenemase activity due to the high value carbapenems have as second or third line antibiotic (Bush, 2010; Tzouvelekis et al., 2012). Carbapenemases are enzymes hydrolyzing the β-lactam ring of lactam antibiotics (Jeon et al., 2015). Many carbapenemases recognize almost all hydrolyzable lactams and most are resilient against inhibition by common lactamase inhibitors (Queenan and Bush, 2007).

Technically, MALDI-TOF MS is not the first choice for studies of degradation kinetics for several reasons:

MALDI-TOF MS is not a fully quantitative method as is, for example, LC-MS (see below). The use of internal standards for quantification results in semi-quantitative results at best. This means that for the estimation of metabolic rates, ratios of peak heights or peak areas are considered and not true concentration values. Therefore, any antibiotic metabolization has to be progressed if not nearly completed before a positive signal can be unambiguously concluded. Fortunately, the hydrolysis is achieved rapidly with most carbapenemases, this is within less than an hour given the bacterial inoculum is sufficiently dense (Mirande et al., 2015).

Many molecules tend to form not only singly protonated ions during the ionization phase but also form sodium and potassium adduct peaks (and possibly di-sodium adduct peaks, sodium/potassium adduct peaks, water abstracted peaks, etc.). In consequence, a single, pure antibiotic may be detected as a series of several peaks the number of which often is even increased when the compound forms fragments during the acceleration phase, for example, through the loss of a carboxy moiety. In a mass spectrum of a degradation assay a multitude of peaks needs to be considered to fully interpret the data. This makes multiplexing, using combinations of antibiotics in a single assay, rather complicated because peaks may overlap when more than one compound is analyzed at a time, like Hooff et al. (2012) proposed by using bacterial lysates instead of vital bacterial cells.

Especially the last point complicates the interpretation of mass spectra. For example, while the [M + H]^+^molecular peak of ertapenem appears at *m/z* 476.6, the corresponding adduct peaks at *m/z* 498.7, 514.6, 520.6, 536.7, and 542.6 Da for [M + Na]^+^, [M + K]^+^, [M + 2Na]^+^, [M + Na + K]^+^, and [M + 3Na]^+^, respectively (Sparbier et al., 2012). Peaks representing the different hydrolysis products were found at 472.6, 488.6, 494.7, 516.6, 538.7, and 554.7 Da, corresponding to the [M + H_2_O-CO_2_ + Na]^+^, [M + H_2_O-CO_2_ + K]^+^, [M + H_2_O + H]^+^, [M + H_2_O + Na + H]^+^, [M + H_2_O + 2Na + H]^+^, and [M + H_2_O + Na + K + H]^+^, respectively. This complicates predictions about the presence and relative intensity of the various analyte related peaks, which in turn complicates the distinction of biological activity from physico-chemical effects. With multiple peaks per analyte in the mass spectra some of the peaks are found close to each other and potentially overlap (Carvalhaes et al., 2013). Hence it is very important to have the system accurately calibrated to allow for the correct interpretation of observed peaks as degradation products.

These issues are specific to MALDI-TOF MS and with other, truly quantitative analytical procedures, such as HPLC or LC-MS, these drawbacks would possibly be avoided (Peaper et al., 2013). With chromatographic methods generally one peak of the native compound and one of the hydrolyzed product occur in chromatograms, with both compound being independently quantifiable over a wide range of concentrations.

Several antibiotics are not very stable in aqueous solutions complicating the distinction between spontaneous degradation in a negative control (antibiotic plus inactivated bacterial cells, when done correctly) and degradation due to enzymatic activity. A fully quantitative method possibly can also distinguish microbial activity from (spontaneous) chemical degradation with more confidence, in particular, when the microbial degradation results in different products compared to chemical breakdown. Negative control strains lacking the relevant lactamase genes need to be included in all studies.

Despite these limitations, the successful application of the assay has been documented for a wide array of bacterial species [*Acinetobacter* sp. (Kempf et al., 2012; Alvarez-Buylla et al., 2013; Carvalhaes et al., 2013); *Bacteroides* (Johansson et al., 2014b); *Pseudomonas* (Carvalhaes et al., 2013; Lee et al., 2013); and Enterobacteriaceae (Sparbier et al., 2012; Jung et al., 2014b; Oviaño et al., 2016a; Calderaro et al., 2017; Choquet et al., 2018]; with different antibiotics as substrate [ampicillin (Jung et al., 2014b); cefotaxime and ceftazidime (Oviaño et al., 2014, 2017a; Li et al., 2017); ertapenem (Burckhardt and Zimmermann, 2011; Carvalhaes et al., 2013; Hoyos-Mallecot et al., 2014; Johansson et al., 2014a); imipenem (Knox et al., 2014; Chong et al., 2015; Lasserre et al., 2015; Ghebremedhin et al., 2016); meropenem (Hrabák et al., 2011; Wang et al., 2013a; Kawamoto et al., 2018)]; with inoculae collected from positive blood-cultures (Carvalhaes et al., 2014; Johansson et al., 2014b; Jung et al., 2014b; Oviaño et al., 2014) or urine samples (Oviaño et al., 2017b) and with isolates possessing different lactamases.

Differentiation of particular lactamases is possible to some degree, for example, through the inhibition of metallo-lactamases with EDTA (Hoyos-Mallecot et al., 2014) or phenyl boronic acid and 2,6-pyridinedicarboxylic acid) for strains with KPC and NDM, respectively (Monteferrante et al., 2016). The incubation time required for a clear positive result (considering the limitation in quantification accuracy mentioned above) ranges from less than 30 min (Lasserre et al., 2015) to 3–4 h. In most studies, an incubation time of maximally 2 h yielded positive results. The differences in incubation durations likely are caused by variations in inoculum densities (Mirande et al., 2015), with high densities leading to faster results with a significant degradation within minutes in some strains. As the hydrolysis of lactams is a kinetic process, the choice of reaction time is critical to the sensitivity of the test. In case of inconclusive results, an extension of the incubation time is possible because for the MALDI-TOF MS analysis only a fraction (generally 1 μL per spot) of the inoculum is required (Ghebremedhin et al., 2016).

The need to extend incubation time has also be reported for isolates carrying OXA-type carbapenemases, in particular, in *Acinetobacter* sp. (Oviaño et al., 2016b; Rapp et al., 2018). Lee et al. (2013) incubated respective strains for 12 h to achieve 100% sensitivity. Papagiannitsis et al. (2015) propose the addition of ammonium hydrogen carbonate to improve the reactivity of OXA-48. The incubation medium may also affect the outcome. For example, Ramos et al. (2016) found false negative results for *Acinetobacter baumannii* harboring OXA-type carbapenemases specifically when grown on MacConkey agar for unknown reasons. With Enterobacteriaceae carrying OXA-type carbapenemases, on the other hand, rapid antibiotic hydrolyzation has been observed (Sauget et al., 2014) or could be overcome by exchanging the substrate ertapenem with temocillin (Oviaño et al., 2016a).

The combination of the chosen substrate antibiotic and the particular lactamase apparently is critical for the performance of the test. Knox et al. (2014) reported false negative results for some Enterobacteriaceae (*Proteus* sp. with VIM-1, NDM) when using imipenem as substrate while in studies using meropenem, Enterobacteriaceae with these lactamases produced positive results (Hrabák et al., 2012; Vogne et al., 2014).

To increase reproducibility, a standardization of the assay is desirable (Choquet et al., 2018). Respective attempts include, for example, a cell lysis and protein extraction step (Monteferrante et al., 2016). Several additives to the incubation suspension have been proposed to stabilize the antibiotic or to protect the enzymatic activity, such as zinc sulfate, sodium dodecyl sulfate, ammonium hydrogen carbonate (Studentova et al., 2015; Knox and Palombo, 2017; Oviaño and Bou, 2017).

A number of points need to be considered with respect to result interpretation:

• A lack of degradation of one antibiotic does not necessarily yield information on the possible lack of degradation of another compound. In consequence, each antibiotic theoretically needs to be tested separately or in multiplexed assays with the limits listed above.
• A lack of observed degradation does not allow to conclude on susceptibility not to speak of an MIC. It simply does not show degradation activity and may possess another resistance mechanisms, for example, a mutation in porins, preventing the antibiotic to enter the cell (Livermore, 2001; Vila et al., 2007; Hao et al., 2018).
• Antibiotic degradation activity is subject to transcriptional regulation and the degradation activity per cell can vary considerably. Possibly, exposure of naïve cells to an antibiotic results in a lag-phase during which activity is low and only when the expression of enzyme is upregulated, the degradation rate increases, this is, degradation of antibiotics is related to exposure (Palmer et al., 2018).

The potential shortcomings of the assay are supposedly outbalanced by the simplicity, the low costs (Lasserre et al., 2015), and, more important, the rapidity with which (positive) results are obtained, although in a laboratory workflow with high-throughput identification, instrument availability may be a limiting factor to run degradation assays. The fact that for individual isolates inconsistent results have been obtained, is more often rather a matter of the complexity of microbiology than due to weaknesses of the assay itself and conventional tests face the same or similar limitations.

A software module to evaluate spectra acquired in degradation assay, MBT STAR^®^-BL, has been developed and commercialized by Bruker Daltonik (Bremen, Germany), together with a sample preparation kit (MBT STAR^®^ -Carba IVD Kit). This software makes the manual and hence tedious identification of peaks related to native and degraded antibiotics obsolete and yields a result through semi-automated data processing and has been tested in several clinical studies (Papagiannitsis et al., 2015; Oviaño et al., 2017a; Kawamoto et al., 2018; Rapp et al., 2018).

In parallel to the assays based on MALDI-TOF MS, a similar assay that makes use of LC-MS for the quantification of antibiotic degradation kinetics has been developed. The fully quantitative technique allows the detection of minor changes in antibiotic concentration (Peaper et al., 2013), in particular when internal standards are used (Wang et al., 2013b). As substrate antibiotics, meropenem (Foschi et al., 2015), ertapenem (Peaper et al., 2013), and imipenem (Kulkarni et al., 2014) have been deployed to detect carbapenemase (OXA-48, IMP, NDM, and KPC) activity in Enterobacteriaceae (Huber et al., 2016; Tadros et al., 2018), *Acinetobacter baumannii* (Lin et al., 2016), and other taxa. The technique can potentially be applied equally widely as the MALDI antibiotic degradation assay, except for the same limitations discussed above. The higher accuracy of LC-MS analysis compared to semi-quantitative MALDI-TOF MS is traded by higher costs for instrumentation and longer run-time of analyses. Since antibiotic hydrolysis acts rapidly for most carbapenemases, detection of small changes in antibiotic concentrations are not necessarily required for a reproducible and robust test. Therefore a higher accuracy of LC-MS may not offer significant advantages for routine diagnostics.

Besides tests based on MS, a number of other tests have been recently developed delivering similar results and fill the same niche as the MALDI-TOF MS assay in a clinical laboratory. The phenotypic detection of hydrolytic activity by a colorimetric test has been developed (Dortet et al., 2012; Österblad et al., 2016; Surre et al., 2018) and commercialized as RAPIDEC^®^ CARBA NP (bioMérieux, Marcy l’Étoile, France) and as beta-LACTA test (Bio-Rad, Marnes-la-Coquette, France). The test is based on a change in pH induced by the hydrolysis activity that induces the color change of an indicator. The simple procedure involves the suspension of an isolate and observation of color changes at defined time-points. Only few studies exists that compare the performance in a comprehensive manner (Knox et al., 2014; Chong et al., 2015; Dortet et al., 2018b; Zhou et al., 2018). It seems that differences in performances of individual tests vary from study to study but generally only to a limited extent. Further molecular, biochemical, and non-molecular methods are available or under development (Bialvaei et al., 2016; Aguirre-Quiñonero and Martínez-Martínez, 2017; Decousser et al., 2017), generally comprising specific tests for individual types of lactamases or individual taxa, for example, the immune-chromatographic detection of particular types of carbapenemases (Glupczynski et al., 2016). Therefore, the clinical microbiologist can chose the test that best suits the requirements and possibilities under local settings.

## Detection of Microbial Growth

**FIGURE 6:**
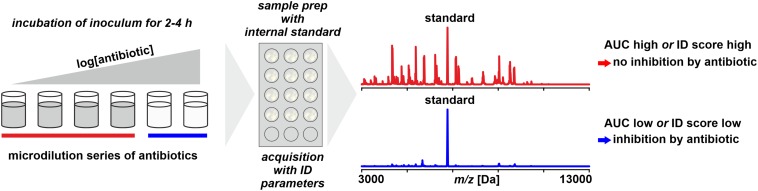
Monitoring microbial growth in the presence of antibiotics by MALDI-TOF MS. As benchmark for positive growth thresholds for either area under the curve (AUC) statistics derived from receiver–operator-characteristics are or identification scores as for routine identification are used.

A different approach is the monitoring of microbial growth by MS of whole cells (Arnold et al., 1999). This technique is possibly the one that comes closest to conventional AST and can yield information on an isolates susceptibility rather than its resistance (Figure 6). Several methods have been proposed of which the one commercialized as MBT-ASTRA (Bruker Daltonik, Bremen, GER) seems the most promising one.

The growth of an isolate – or the lack thereof – is monitored by incubating a low-density inoculum in the presence of an antibiotic and, after a predefined incubation time, harvesting the total of the cells and performing a spectrum acquisition by MALDI-TOF MS (Lange et al., 2014). To allow a quantitative interpretation, an internal standard (for example, RNAse B) is added to the sample. The data interpretation is based on an Area Under the Curve (AUC) statistics, a tool that is widely used in Receiver-Operator Characteristics (ROC) to evaluate the accuracy of diagnostic tests (Swets, 1988; Faraggi and Reiser, 2002). It basically compares the number and intensity of peaks recorded in a negative control (without antibiotics) to those in a treated sample. The more the growth is inhibited, the more the ratio is shifted and this shift is interpreted by a proprietary software (Sparbier et al., 2016).

The method is applicable to all microorganisms growing in homogeneous suspension, including mycobacteria (Ceyssens et al., 2017), anaerobes (*Bacteroides fragilis*; Justesen et al., 2018), yeasts (Vatanshenassan et al., 2018), and positive blood cultures (Jung et al., 2016), indicating that there is no principle obstacle for all types of microorganisms, except probably for molds and very slowly growing species.

However, limitations of the method arise from several issues: firstly, when only a single concentration of antibiotics is tested, this needs to be optimized to yield meaningful results to direct therapy (Maxson et al., 2017). Secondly, the measurement inevitably is an end-point measurement. This means, in case of inconclusive results, for example, due to an unexpectedly delayed growth, the incubation time cannot be simply extended as this is done in conventional tests like broth microdilution assays. Thirdly, the possible effect of inoculum density requires further attention because the success of the test is based on choosing an inoculum that is below but close to the detection limit of WCMS to yield a positive result (a spectrum allowing identification indicating growth) after just a few cell doublings during the short incubation time of 3–4 h. This would be overcome, of course, by an extension of the incubation time but this would negate the advantages compared to conventional phenotypic tests. Globally, a detection limit for bacteria is estimated at 10^4^ cells per sample spot (Li et al., 2009) corresponding to 10^7^ cells/mL for 1 μL sample aliquots for MALDI analyses. But the limit of detection expectedly varies considerable between taxa due to differences in cell size and shape, thus possibly requiring taxon-specific guidelines for inoculum preparation based on simple measurements such as optical density.

An advancement of the method is the incubation for 3h of the bacterial suspension in micro-droplets of a few micro-liters directly on the MALDI-target (Idelevich et al., 2018). This allows a lay-out with multiple antibiotic concentrations to achieve a test closer to CLSI (2018b) or EUCAST (2013) guidelines than with testing only a single concentration. Categorization is based on standard MALDI Biotyper identification scores, where at <1.7 (median of three replicates), the isolate was considered as susceptible, or more correct, inhibited by the chosen antibiotic concentration. The same method was also applied to positive blood cultures, however, only with a single antibiotic concentration (Idelevich et al., 2018). The major critical step in this method is the removal of growth medium after the predefined incubation time without a loss of bacterial cells that would necessarily distort the result. Idelevich et al. (2018) proposed to do this manually with a filter paper by making use of capillary forces. Desirably, this step is to be automated in some way to avoid bias due to variability in operators’ skills and vigilance. A test layout in full agreement with EUCAST guidelines was recently proposed by Correa-Martínez et al. (2019) to screen Enterobacteriaceae for resistances due to ESBL and AmpC lactamases. It is a further improvement of the micro-droplet assay with a full range of antibiotic concentrations (0.25–512 μg/mL) and lactamase inhibitors (clavulanic acid). As cut-off indicating microbial growth after an incubation of 4 h, an identification score of ≥2.0 (Freiwald and Sauer, 2009) is considered and the MIC as the highest concentration yielding a score < 2.0.

Two of the three limitations discussed above also apply to the micro-droplet assay, this is the manual removal of liquid medium and the uncertainty possibly associated with the inoculum density. Both may be overcome by techniques such as fixation of bacterial cells to the surface (Stevens and Jaykus, 2004; Park et al., 2011) to reduce the risk of accidental loss of biomass and facilitate the removal of liquid medium. Inoculum density could possibly be optimized by providing taxon-specific requirements in terms of easily determined optical density. For the impossibility to extend incubation time once a sample has been prepared by adding matrix solution – and by this killing microbial cells – it is difficult to imagine an efficient solution.

A particular variant of an assay monitoring growth suggested by Demirev et al. (2013) and Sparbier et al. (2013) is based on the supplementation of the growth medium with stable-isotope labeled amino acids (generally lysine ^13^C/^15^N labeled to defined degrees). Isolates unaffected by the presence of antibiotics incorporate the labeled lysine in *de novo* synthesized proteins, resulting in gradual mass shifts of individual peaks in mass spectra (Jung et al., 2014a). Although applied widely in proteome research (Chahrour et al., 2015), the price of commercially available labeled compounds is most likely prohibitive for an implementation of this technique in routine diagnostics.

An assay that targets not the growth, i.e., increase in total biomass, but changes in protein expression profiles of *Candida albicans* isolates exposed to a gradient of concentration of antibiotics was developed by Marinach et al. (2009). After an incubation for 15 h in serial dilutions of fluconazole (0.125–128 mg/mL) and a washing step, aliquots were prepared and spectra acquired by MALDI-TOF MS. In the spectra, differences in peaks’ presence and intensity was observed and a “minimal profile change concentration,” defined as the lowest drug concentration that induced a significant change in the protein peak profiles.

This principle was extended to further *Candida* species and *Aspergillus* sp. and the inclusion of composite correlation index statistics (De Carolis et al., 2012). This allowed to identify MICs in a concentration range of caspofungin (0.008–64 μg/mL). A simplification was proposed by Vella et al. (2013), that is based on only three concentrations at breakpoint levels and an incubation for only 3 h, and evaluated with a set of 80 *C. glabrata* isolates (Vella et al., 2017). Testing further species and antibiotics revealed, however, that reproducibility varied between species and antibiotics (Saracli et al., 2015), suggesting more studies to standardize the assay.

## Mass Spectrometry Based Ast Techniques in the Wider Context

Different tracks have been followed to detect antibiotic susceptibility and resistance by MS, some of which are commercialized. From a wider angle, however, MS is only one player among others in recent developments of susceptibility and resistance testing, respectively (van Belkum and Dunne, 2013). The underlying motivation is either to reduce the cost of individual tests, to increase the accuracy of the result or to shorten the analysis time from more than 6 h to 2 h or less – or ideally all three (Leonard et al., 2018; Maugeri et al., 2019).

One direction that is followed is the application of modern micro- and nano-technologies, like laser scatter (Hayden et al., 2016) or microfluid (Jiang et al., 2016) technologies. These techniques can be considered as phenotypic susceptibility tests with a higher sensitivity and a shorter time to result, but are not yet widely used. On the other hand, tests based on next-generation sequencing technologies for clinical microbiological diagnostics primarily target antibiotic resistance by identification of respective genes. Whole-genome sequencing and metagenomics have high potential (Forbes et al., 2017; Quainoo et al., 2017) but for reliable antimicrobial susceptibility testing by whole genome sequencing more data and tests are supposedly needed before it can begin to replace conventional tests (Ellington et al., 2017).

The near future will show which of these diverse tests have the potential to, at least partly, replace conventional susceptibility and resistance testing. With today’s methods, such as disk diffusion or broth microdilution, a large amount of data is constantly compiled and analyzed to define breakpoints and to issue recommendations for treatment (CLSI, 2018a; EUCAST, 2019). The inevitably less comprehensive database for any new method likely constitutes an obstacle for their widespread introduction, in particular, in low income settings where resources for substantial investments in instrumentation (and expertise) are limited (Ombelet et al., 2018).

## Concluding Remarks

For most of the methods for antibiotic susceptibility and resistance testing by MS discussed above, an implementation in routine workflows in a microbiology laboratory has not yet been achieved and there are good reasons to believe that particular methods will remain primarily of academic interest. Yet, only 15 years ago the idea to identify microorganisms in high numbers in clinical routine by MALDI-TOF MS after very basic sample preparation was generally granted with a patronizing smile (own experience, unpublished). It sounded just way too optimistic to most microbiologists – which wasn’t the case at all, as we know today. Therefore, some of the methods for antimicrobial susceptibility or resistance testing by MS discussed here may become part of routine microbiological diagnostics in clinical laboratories in the near future, even if the constraints we were seeing today seem to be difficult to overcome.

## Author Contributions

All authors listed have made a substantial, direct and intellectual contribution to the work, and approved it for publication.

## Conflict of Interest

MW and AB are employees of BioMérieux, a vendor of diagnostic systems for clinical microbiology, including mass spectrometry and antibiotic susceptibility testing. The reviewer WG declared a past co-authorship with the authors AB and MW to the handling Editor.
